# Consistency of associations of systolic and diastolic blood pressure with white matter hyperintensities: A meta-analysis

**DOI:** 10.1177/17474930211043364

**Published:** 2021-09-10

**Authors:** Imogen Wilkinson, Alastair John Stewart Webb

**Affiliations:** Centre for Prevention of Stroke and Dementia, University of Oxford, Oxford, UK

**Keywords:** Hypertension, blood pressure, meta-analysis, white matter hyperintensities

## Abstract

**Background:**

White matter hyperintensities are the commonest manifestation of cerebral small vessel disease, associated with stroke, functional impairment, and cognitive decline. They are commonly preceded by hypertension, but the magnitude and clinical importance of this association is unclear.

**Aims:**

Quantify the relationship between blood pressure and white matter hyperintensities across studies.

**Methods:**

PubMed and EMBASE were searched for studies reporting associations between concurrent or historic blood pressure and white matter hyperintensities. Beta coefficients from linear models were extracted, whether standardized, unstandardized, unadjusted or adjusted for age, sex, and cardiovascular risk factors. Beta-coefficients were combined by fixed and random effects meta-analysis, combining standardized beta-coefficients or unstandardized coefficients measured by consistent methods.

**Results:**

Twenty-five of 3230 papers were eligible, including 53,392 participants. Systolic blood pressure was significantly associated with white matter hyperintensity volume (WMHV) after maximal adjustment (standardized beta 0.096, 95%CI 0.06–0.133, p < 0.001, I^2 ^= 65%), including for concurrent readings (b = 0.106, p < 0.001) or readings five years previously (b = 0.077, p < 0.001), and for younger or older populations (mean age < 65: b = 0.114; >65 b = 0.069). Unstandardized, adjusted associations were similar for raw WMHV, log-transformed WMHV, or WMHV as percentage of intracranial volume. Unadjusted associations with systolic blood pressure (SBP) were greater (standardized beta = 0.273, 0.262–0.284, p < 0.0001). However, while associations with DBP were weaker than SBP (standardized beta = 0.065, p < 0.001), they were minimally affected by adjustment for age.

**Conclusions:**

A standard deviation increase in SBP is associated with 10% of a standard deviation increase in WMHV, providing the current best estimate of the potential reduction in progression of white matter hyperintensities expected with good control of blood pressure.

## Introduction

White matter hyperintensities (WMH) of presumed vascular origin are the commonest manifestation of cerebral small vessel disease,^1^ occurring in the majority of patients over 65 years of age and nearly all people by 90 years.^2^ They are associated with up to 30% of strokes and 40% of dementia,^3^ as well as later life refractory depression^4^ and functional impairment.^5^ They are most strongly associated with hypertension,^6^ both systolic blood pressure, particularly in later life and diastolic blood pressure, particularly in mid-life,^6^ reflecting increased arterial pulsatility and increased arterial stiffness.^7^ Intensive treatment of hypertension reduces progression of these markers of vascular aging, and in post hoc analyses of clinical trials, it reduces rates of progression of WMH.^8^ However, the magnitude of the potential benefit in preventing WMH by control of hypertension is unclear, as well as the optimal time of intervention and blood pressure target.^6^

The relationship between SBP or DBP and WMH appears to interact with age. Systolic blood pressure (SBP) increases linearly with age, while DBP initially rises before declining after approximately 55 years of age for men and 58 years of age for women.^7^ This reflects transition from mid-life hypertensive phenotypes characterized by sympathetic overactivity, transitioning to late-life phenotypes characterized by increased arterial stiffness, small vessel rarefraction, and greater arterial pulsatility in aging blood vessels.^9^ This interaction may therefore be reflected in the long-term relationship between SBP or DBP with WMH.

Therefore, we performed a systematic review and meta-analysis to estimate the magnitude of the association between different blood pressure parameters and WMH, stratified by age and time of exposure.

## Methods

### Search strategy

PubMed and EMBASE (via Health Database Advanced Search) were searched from database inception to 1 December 2020, restricted to human studies in English (Supplementary Figure 1; Supplementary data – Search Strategy). Secondary searches were performed in the largest studies in which the primary report did not provide adequate data to enable meta-analysis. Included studies reported a quantitative association between blood pressure level (SBP, DBP, mean blood pressure (MBP), or pulse pressure (PP) and WMH on magnetic resonance imaging (MRI), assessed on either T2 or FLAIR imaging, using validated software for calculation of WMH. Study titles, abstracts, and appropriate full texts were searched sequentially (IW), with all potentially included full text articles independently reviewed by two reviewers (AJSW, IW) in accordance with pre-specified criteria defined in the protocol. Reference lists of relevant studies were also searched. Assessment of study quality for included papers was carried out using the NIH study quality assessment tool.^10^ Publication bias was assessed by funnel plots. The protocol for this search was registered and published, via PROSPERO. The data that support the findings of this study are available from the corresponding author upon reasonable request, and are all available in published journals.

### Data *e*xtraction

The primary extracted outcome was the beta coefficient between blood pressure index and volume of WMH reported in linear models, where WMH volume was reported quantitatively, whether raw, log-transformed, logged, or normalized to the intracranial volume. Standardized and unstandardized beta-coefficients were included, adjusted and unadjusted for covariates. Other variables extracted included demographics of the included population (age, gender, blood pressure, arterial stiffness, comorbidities), study characteristics (prospective vs. retrospective, cohort vs. case control vs. trial), inclusion/exclusion criteria, interval between BP measurement and MRI imaging, method of quantification of WMH, and details of analytical models (model type, univariate vs. multivariate analysis, covariates included). Measures of uncertainty of variables were extracted where available, including standard deviation (SD), standard error or interquartile range. Where possible, unstandardized beta coefficients were converted to standardized via multiplication of the coefficient by the ratio of SD of BP to SD of white matter hyperintensity volume (WMHV) and vice versa. Where necessary, SD of WMHV was estimated from the interquartile range, including for logged WMHV, by interquartile range/1.35, as recommended by the Cochrane Handbook.^11^ Where the standard error of the beta-coefficient was not reported, it was estimated from reported beta, p-value, and degrees of freedom of the model (n−1-number of covariates), or else it was imputed by the ratio of the study size of the study in question to the size of studies where an inverse variance could be determined.

### Meta-analysis

Beta coefficients were combined by fixed and random effects meta-analysis, weighted by the inverse variance.^12^ Heterogeneity was assessed via I^2^ statistics and Χ^2^ test. The primary analysis was a meta-analysis of standardized beta-coefficients adjusted for age, gender, and cardiovascular risk factors, including results with at least adjustment for age and gender. Further meta-analyses were performed including unadjusted, standardized beta-coefficients and non-standardized beta-coefficients, unadjusted and adjusted, stratified by whether WMH were logged or calculated as proportion of intracranial volume.

## Results

A total of 3230 papers were identified in the primary search, and 578 in secondary searches. Following screening of titles, 1198 papers were reviewed as abstracts and 786 papers were reviewed in full, with 25 papers eligible for inclusion. Standardized beta coefficients were reported or could be calculated in 12 papers (13 populations) for SBP, nine papers for DBP, three papers for MBP, and two papers for PP. In total, there were 53,392 participants, with participant numbers ranging from 56 individuals to 37,026 individuals (Supplementary Table 1). The mean age ranged from 31.7 to 85.8, while the mean WMHV ranged from 0.012 cm^3^ to 13.9 cm^3^.

Of the 25 included papers, quality varied from poor to high, with the majority of papers of moderate quality (Supplementary Table 2). There was limited evidence of publication bias (Supplementary Figure 2), with evidence for reduced reporting of small studies with null or negative associations, but not sufficient to have a significant impact on the summary estimates.

**Figure 1.**
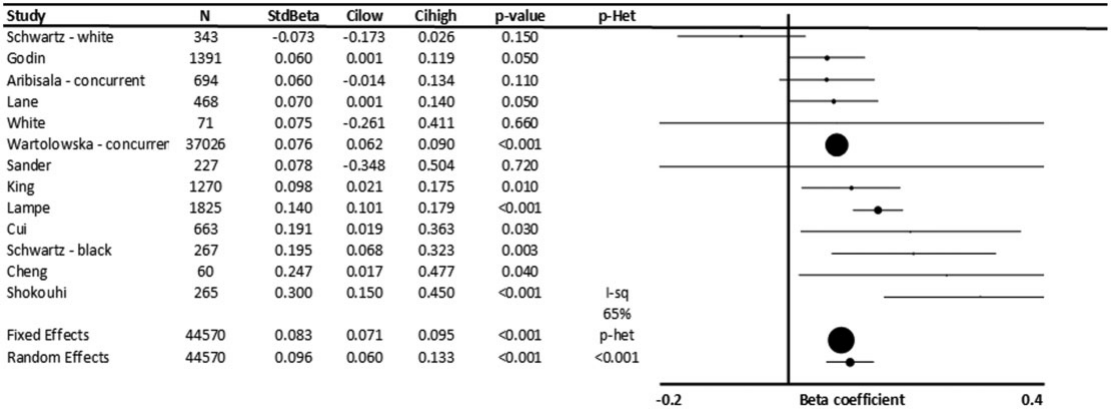
Standardized association between SBP and white matter hyperintensity volume (WMHV). Results are shown for individual studies reporting standardized beta-coefficients, with the maximally adjusted value from each study shown, with all reports adjusted for at least age and sex. Results are combined by fixed and random effects meta-analysis, weighted by the inverse variance, with heterogeneity presented as I^2^ statistics (I-sq), and the p-value for heterogeneity (p-het) determined by chi-squared test. StdBeta: standardized beta; N: number.

In 12 populations including 44,570 people, across all studies, increased systolic blood pressure was significantly associated with increased severity of WMH (p < 0.001, Figure 1).^6,13–20^ In univariate comparisons, without adjustment for age, the standardized beta-coefficient was 0.273 (Supplementary Figure 3) reducing to a mean 0.096 standardized beta-coefficient after adjustment for cardiovascular risk factors, partially reflecting the strong association between age and SBP. This represents an approximately 10% SD increase in WMH per SD increase in SBP. This was similar even when excluding the largest study (Supplementary Figure 4).^6^

**Figure 2.**
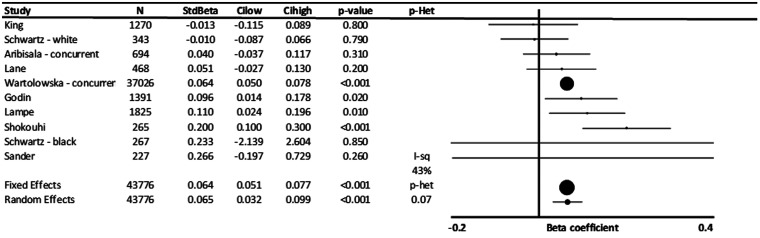
Standardized association between diastolic blood pressure and white matter hyperintensity volume (WMHV). Results are shown for individual studies reporting standardized beta-coefficients, with the maximally adjusted value from each study shown, with all reports adjusted for at least age and sex. Results are combined by fixed and random effects meta-analysis, weighted by the inverse variance, with heterogeneity presented as I^2^ statistics(I-sq), and the p-value for heterogeneity (p-het) determined by chi-squared test. StdBeta: standardized beta; N: number.

Standardized beta-coefficients for the association between DBP and WMH were weaker (β = 0.065, p < 0.001), although this difference was not significant (Figure 2). However, the standardized beta-coefficient between DBP and WMHV was similar before and after adjustment for age and other risk factors, reflecting the weaker relationship between DBP and age (Supplementary Figure 3). In the limited studies reporting standardized beta-coefficients for MBP and PP, there was no clear association between blood pressure and WMHV (Supplementary Figure 5).

**Figure 3.**
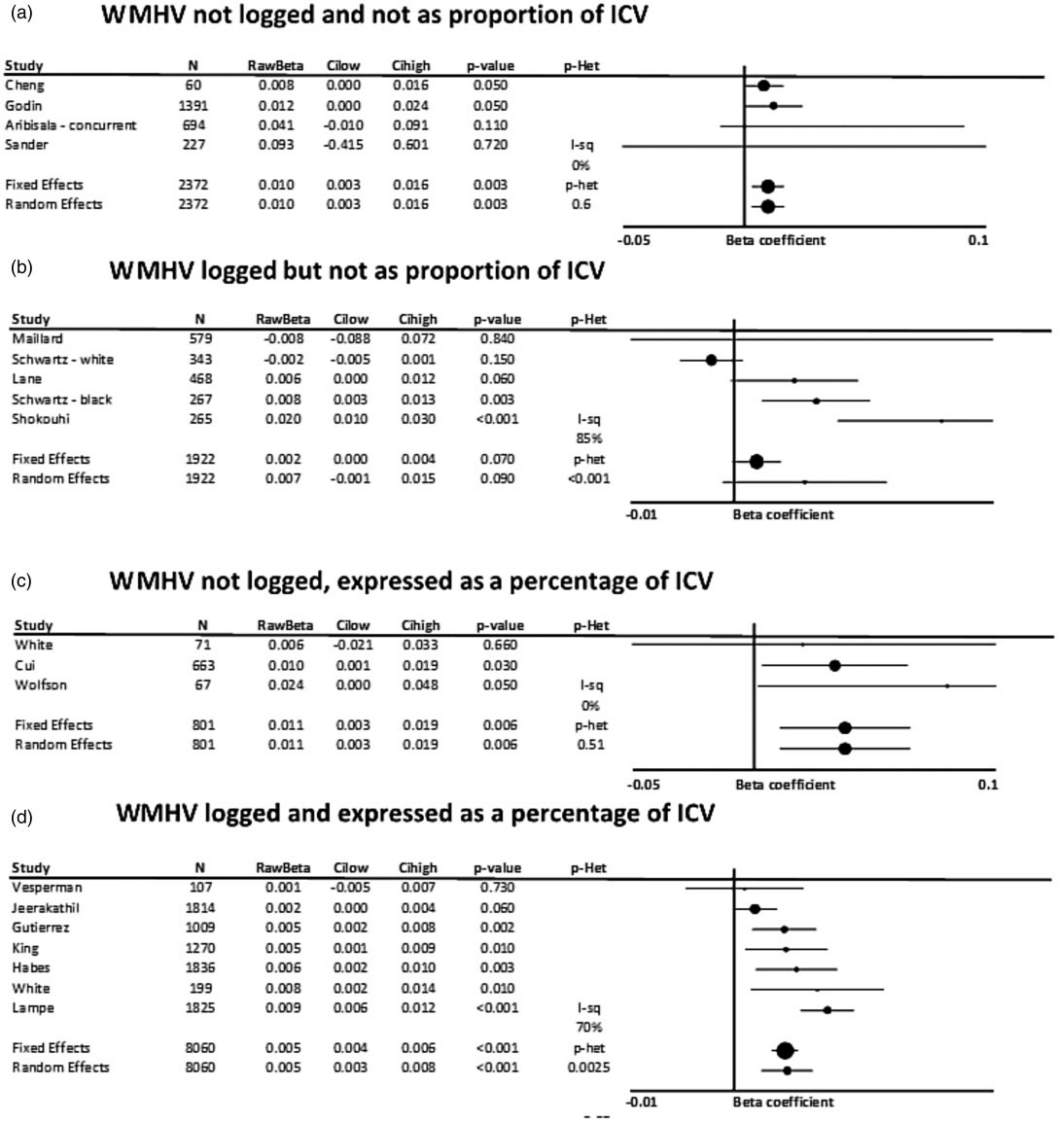
Unstandardized, adjusted associations between systolic blood pressure and white matter hyperintensity volume (WMHV), stratified by whether WMHV were log-transformed or expressed as a percentage of intracranial volume. Results are shown for studies reporting standardized beta-coefficients, with the maximally adjusted value from each study shown, with all reports adjusted for at least age and sex. Results are combined by fixed and random effects meta-analysis, weighted by the inverse variance, with heterogeneity presented as I^2^ statistics(I-sq), and the p-value for heterogeneity (p-het) determined by chi-squared test. StdBeta: standardized beta; N: number; ICV: intracranial volume. (a) WMHV not logged and not as proportion of ICV. (b) WMHV logged but not as proportion of ICV. (c) WMHV not logged, expressed as a percentage of ICV. (d) WMHV logged and expressed as a percentage of ICV.

In papers reporting unstandardized beta-coefficients, or where this could be estimated from standardized beta-coefficients, SBP was consistently associated with WMHV, whether WMH were log-transformed, divided by the intracranial volume or both (Figure 3). This corresponded to a 0.01 cm^3^ greater WMH per 1 mmHg increase in SBP in non-transformed associations, or a 0.011% increase in the proportion of intracranial volume occupied by WMH. However, there was significant heterogeneity in the five studies reporting results by log of WMH, limiting conclusions about the magnitude of the effect.

**Figure 4.**
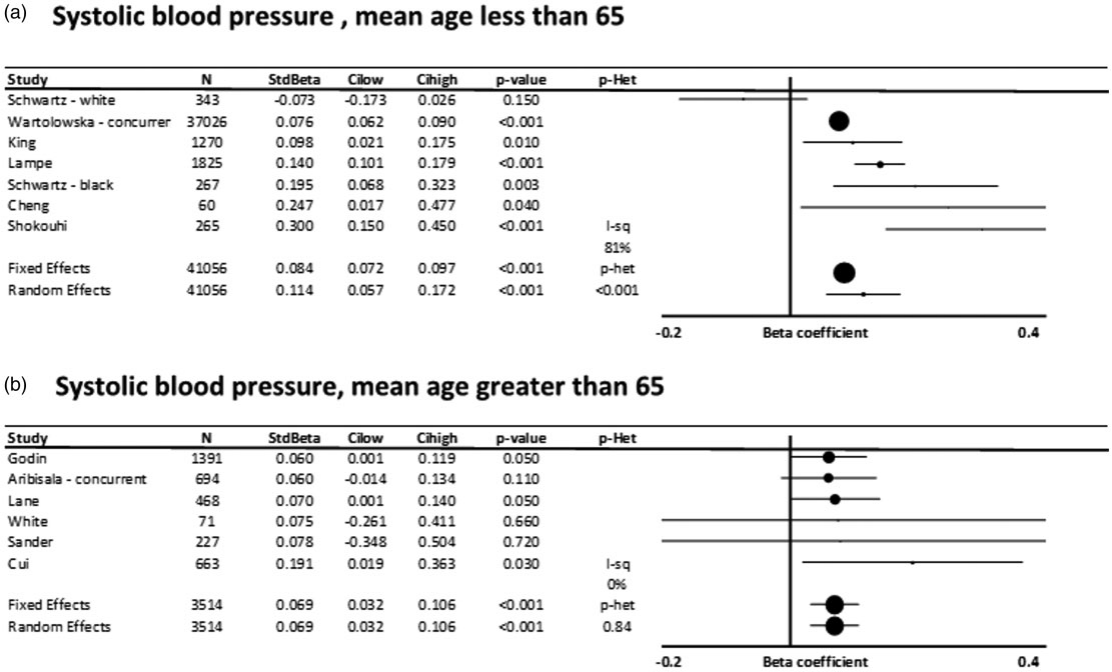
Standardized, adjusted associations between systolic blood pressure and white matter hyperintensity volume (WMHV), stratified by a mean age of greater than or less than 65. Results are shown for individual studies reporting standardized beta-coefficients, with the maximally adjusted value from each study shown, with all reports adjusted for at least age and sex. Results are combined by fixed and random effects meta-analysis, weighted by the inverse variance, with heterogeneity presented as I^2^ statistics(I-sq), and the p-value for heterogeneity (p-het) determined by chi-squared test. StdBeta: standardized beta; N: number. (a) Systolic blood pressure, mean age less than 65. (b) Systolic blood pressure, mean age greater than 65.

**Figure 5.**
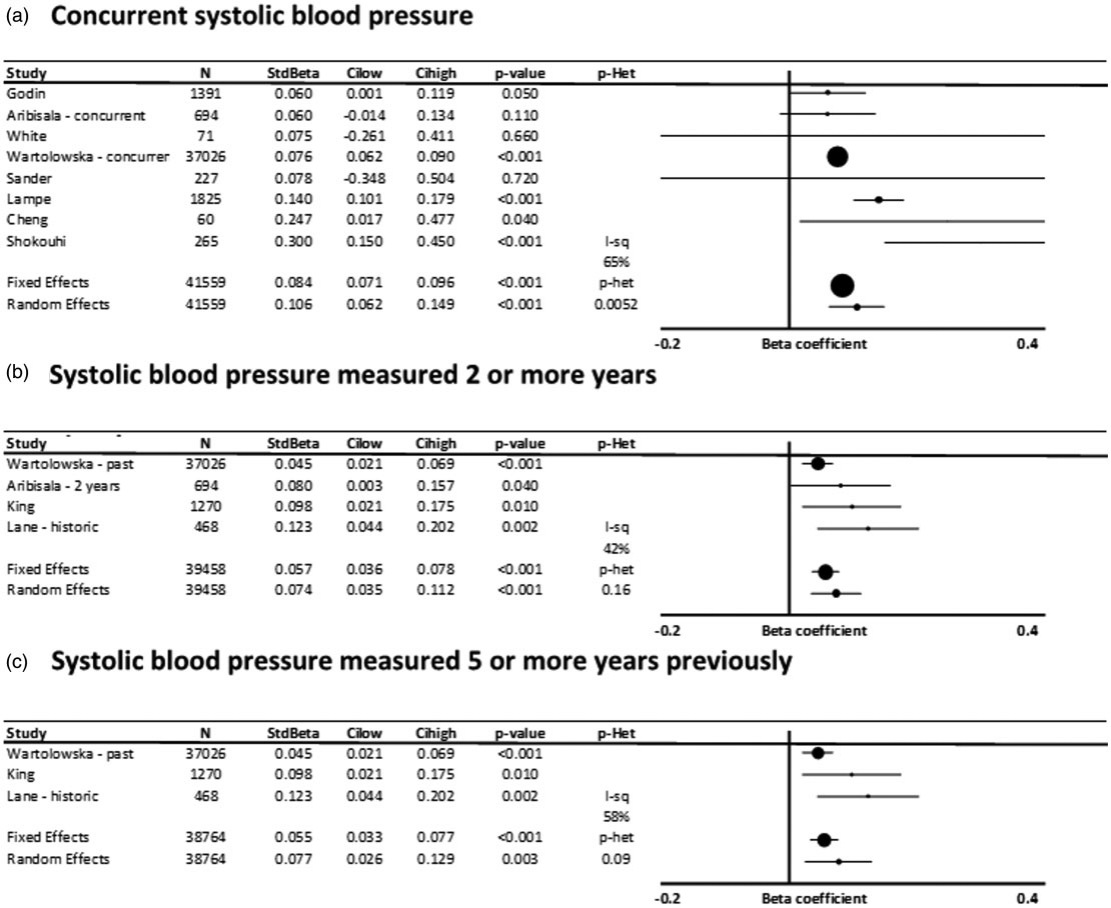
Standardized, adjusted associations between systolic blood pressure and white matter hyperintensity volume (WMHV), stratified by time interval between blood pressure measurement and MRI imaging. Results are shown for individual studies reporting standardized beta-coefficients, with the maximally adjusted value from each study shown, with all reports adjusted for at least age and sex. Results are combined by fixed and random effects meta-analysis, weighted by the inverse variance, with heterogeneity presented as I^2^ statistics(I-sq), and the p-value for heterogeneity (p-het) determined by chi-squared test. StdBeta: standardized beta; N: number. (a) Concurrent systolic blood pressure. (b) Systolic blood pressure measured two or more years. (c) Systolic blood pressure measured five or more years previously.

Associations between SBP and WMH were not significantly different in populations with a mean age below 65 (Figure 4) compared to populations with a mean age greater than 65 but with greater heterogeneity between studies (Supplementary Figure 6), while associations with DBP were similar in younger and older populations, although only significant in the older age group. Associations were also similar in studies reporting concurrent associations compared to associations with blood pressure measured >2 or >5 years previously, for both SBP (Figure 5) and DBP (Supplementary Figure 7).

## Discussion

In a moderate number of studies, including more than 50,000 participants, there was a strong and largely consistent association between systolic blood pressure or diastolic blood pressure with severity of WMH. Overall, an SD increase in SBP was associated with approximately a 10% of an SD increase in WMH, after adjustment for age, sex, and other major cardiovascular risk factors. This effect was consistent with associations reporting unstandardized associations between SBP or DBP and WMHV. There was no significant difference in associations with concurrent or historic blood pressure, and only non-significant trends to a stronger association in younger populations or a stronger association with SBP than DBP.

In the SPRINT-MIND study,^8^ a 14.2 mmHg difference in blood pressure for intensive vs. standard treatment was associated with a reduction in progression of WMH by 0.54cm^3^ over four years, equating to approximately a 10–15% reduction in the population SD of WMH volume at follow-up, although with no change in cognitive decline.^21^ This is a marginally greater magnitude of effect to the association demonstrated in this meta-analysis, whether standardized or unstandardized, but the populations are different and this analysis is cross-sectional rather than assessing the association with progression of WMH. Nonetheless, this confirms that reducing blood pressure results in the expected reduction in WMH, supporting a likely causative relationship between BP and WMH severity.

In this meta-analysis, there was no significant difference between associations of SBP and DBP with WMHV, or between concurrent and historic measures. This is in contrast to limited reports from individual studies suggesting a stronger concurrent association between SBP and WMHV, particularly in the elderly, but a stronger association between historic, mid-life DBP with late life WMHV.^6,16^ This likely reflects the heterogeneity between studies in this meta-analysis. In particular, all studies included a range of patient ages, and the mean age in any specific population is a poor surrogate for estimating the effect in younger vs. older participants. The meta-analysis did demonstrate a non-significantly stronger association between concurrent SBP and WMHV than concurrent DBP and WMHV, which is consistent with the difference in effect size in older participants in other studies.

In unadjusted analyses, there was a much stronger association between SBP and WMHV, as would be expected from the strong linear association between SBP and age,^7^ which is significantly reduced after adjustment for age. This may result in underestimation of the potential effect of controlling SBP in reducing WMHV, as the age-related increase in SBP may still be preventable. In contrast, there was little difference in the association between DBP and WMHV before and after adjustment for age. This is also consistent with the non-linear association between age and DBP, with a rise in DBP until the age of 55, followed by a fall in DBP resulting in no significant impact of adjusting for age.^7^ However, given that the association with DBP is largely independent of age, this may also demonstrate that a greater proportion of preventable WMH may reflect elevated DBP, and may still be of greater significance in younger patients. Unfortunately, the limited number of studies focused only on younger or older patients and reporting DBP, means that this meta-analysis is not powered to test this hypothesis as suggested in large individual studies.^6,16^

This meta-analysis has several limitations. Firstly, there were a relatively limited number of papers that reported sufficient data to be included in the meta-analysis, with many papers reporting qualitative or semi-quantitative outcomes, including from large, seminal cohorts (Framingham, Rotterdam Study, Cardiovascular Health Study). As a result, although some stratification of standardized beta-coefficients for SBP was possible, this was not feasible for other analyses. Secondly, there was a significant variation in the analysis used in different studies, with differences in studies reporting standardized or unstandardized beta coefficients, and whether WMHV was log-transformed or divided by the intracranial volume. Although in some studies it was possible to convert between standardized and unstandardized values, there was frequently insufficient data for this. Furthermore, in many studies it was not clear whether standardized or unstandardized coefficients were reported. However, enough studies were reported for each method of analysis for SBP and DBP to enable a meta-analysis to be performed, although this was not the case for MBP or PP. Ideally, reporting of this type of study should be standardized across studies, with standard outcome measures, reporting of both standardized and unstandardized coefficients, and should follow CONSORT guidelines. Thirdly, the time intervals between measurement of BP and WMH varied significantly in the limited studies that reported historic BP and current WMH, preventing a detailed assessment of any temporal gradient of the effect. Fourthly, there remained significant unexplained heterogeneity between studies, which may well relate to differences in methods of acquiring scans (T2 vs. FLAIR), differences in scanner field (1.5 T vs. 3 T), and differences in methods of quantification of WMH (BIANCA^22^ vs. non open source, in-house methods), but there were too few studies with each method to assess this. Finally, despite broad initial search terms and a secondary search for specific large studies, it is likely that not all reports of the relationship between BP and WMH were identified, as this value may often be reported in full texts of papers where the principal focus of the paper is elsewhere. As such, key terms are not always present in the abstract or title, and may not be identified by a targeted search strategy.

Overall, this study identifies a 10% SD of WMH increase per SD increase in SBP. This strongly covaries with age, while the relationship with DBP was largely unchanged by adjustment for age. The relative effect size was largely consistent across different study designs, populations, and methods of data acquisition, as well as between methods of measurement, and was consistent with effect sizes from control of blood pressure in randomized controlled trials.

## Supplemental Material

sj-pdf-1-wso-10.1177_17474930211043364 - Supplemental material for Consistency of associations of systolic and diastolic blood pressure with white matter hyperintensities: A meta-analysis Click here for additional data file. Supplemental material, sj-pdf-1-wso-10.1177_17474930211043364 for Consistency of associations of systolic and diastolic blood pressure with white matter hyperintensities: A meta-analysis by Imogen Wilkinson and Alastair John Stewart Webb in International Journal of Stroke
