# Supplementary material for: Consistency of associations of systolic and diastolic blood pressure with white matter hyperintensities: A meta-analysis
Source: Int J Stroke. 2021 Sep 10;17(3):291–8. doi: 10.1177/17474930211043364 (PMC8864334; doi:10.1177/17474930211043364)
Supplement: sj-pdf-1-wso-10.1177_17474930211043364 - Supplemental material for Consistency of associations of systolic and diastolic blood pressure with white matter hyperintensities: A meta-analysis [file sj-pdf-1-wso-10.1177_17474930211043364.pdf]

Consistency of associations of systolic and diastolic blood pressure with white matter hyperintensities: a meta-analysis

Imogen Wilkinson BA[†], Alastair John Stewart Webb[†] DPhil

Supplementary Figure 1. Prisma Flow Chart

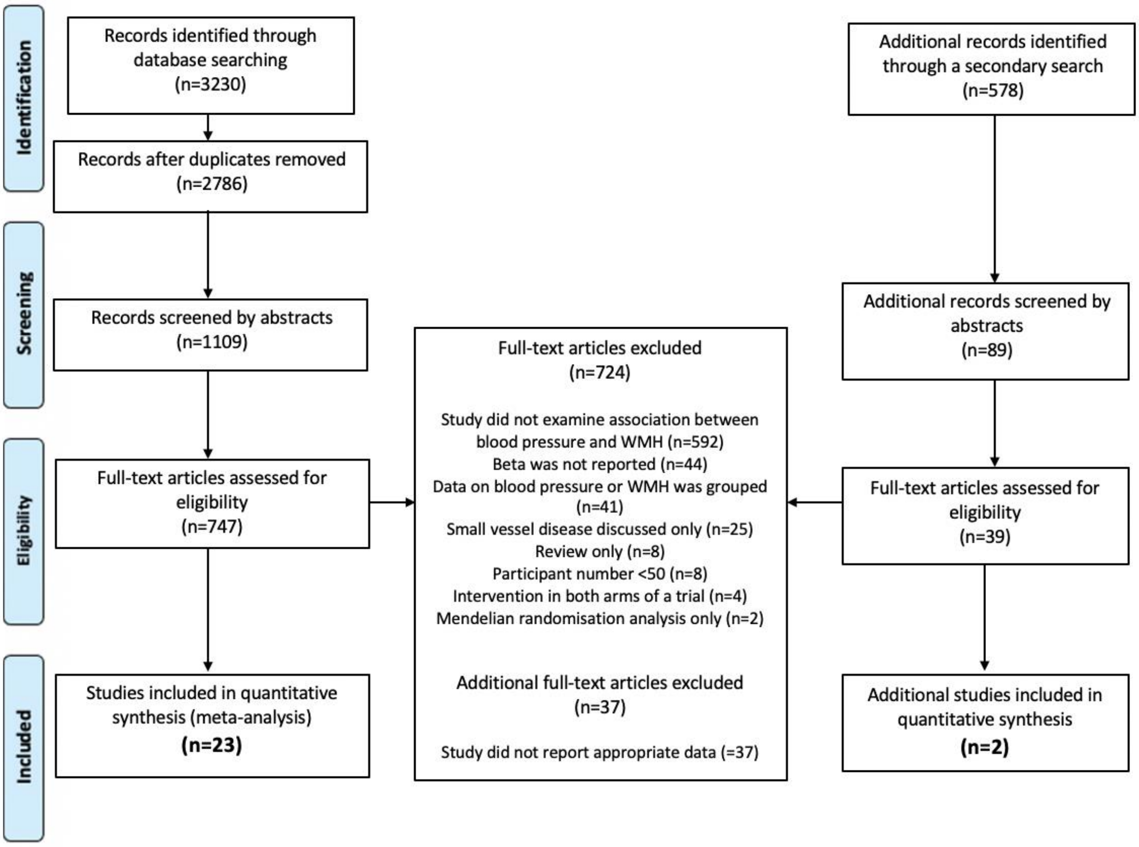

Supplementary Table 1. Characteristics of included studies

Study	N	Mean Age	SD of age	% Male	Mean WMHV (cm^3)	Median WMHV (cm3)
Shi	56	67.8	8.3	40.0	-	10.74
Cheng	60	31.7	6.3	32.0	0.174	-
Wolfson	67	85.8	3.8	39.0	n/a	n/a
White	71	82.1	3.9	40.0	13.9	-
Vesperman	107	64.2	5.9	34.6	2.9	-
Shimizu	133	69.7	3.2	48.0	6.5	-
Alkan	164	60.1	7.9	40.9	0.98	-
White, Jalil	199	81.2	4.1	45.7	n/a	n/a
Sander	227	n/a	n/a	n/a	9.3	-
Heye	264	66.9	11.8	35.0	n/a	n/a
Shokouhi	265	64.0	n/a	35.0	n/a	n/a
Lane	468	70.7	0.7	51.1	3.1	-
Maillard	579	39.2	8.4	40.4	1.7	-
Schwartz	610	62.9	n/a	35.4	-	6.8
Cui	663	68.6	5.7	43.4	-	2.75
Fuhrmann	667	54.6	18.5	n/a	2.15	-
Aribisala	694	69.6	0.8	53.0	0.012	-
Cloonan	809	65.6	14.7	53.0	-	6.25
Gutierrez	1009	64.0	8.0	41.0	n/a	n/a
Tsao	1118	61.0	9.0	44.0	n/a	n/a
King	1270	51.4	9.4	44.3	1.6	-
Godin	1391	72.0	n/a	39.3	5.4	-
Jeerakathil	1814	61.7	9.4	47.0	0.96	-
Lampe	1825	52.5	13.2	43.6	2.835	-
Wartolowska	37026	55.3	7.5	47.0	-	2.796

Supplementary Table 2. Quality assessment of factors that differed between included studies. 0 = no; 1 = yes.

Study	Question 3	Question 5	Question 6	Question 10	Question 12	Question 13	Total
Alkan	0	0	1	0	0	1	2
Aribisala	0	0	1	1	0	0	2
Cheng	1	0	0	0	0	1	2
Cloonan	0	0	1	0	0	1	2
Cui	1	0	1	0	1	1	4
Fuhrmann	1	0	1	0	0	0	2
Godin	1	0	1	1	0	0	3
Gutierrez	0	0	1	1	0	0	2
Habes	0	0	0	1	0	0	1
Jeerakathil	0	0	1	0	1	1	3
King	0	0	1	0	0	1	2
Lampe	0	0	1	0	0	0	1
Lane	0	0	1	1	0	1	3
Maillard	0	0	1	0	0	1	2
Sander	1	0	0	0	1	1	3
Schwartz	0	0	1	1	0	1	3
Shi	0	1	0	0	1	1	3
Shimizu	0	0	0	0	0	1	1
Shokouhi	0	0	1	1	0	0	2
Tsao	0	0	1	0	1	1	3
Vesperman	0	0	0	0	0	1	1
Wartolowska	0	0	1	1	0	1	3
White	1	0	1	1	1	0	4
White, Jalil	0	0	1	0	0	0	1
Wolfson	1	0	1	1	1	0	4

Question 3: Was the participation rate of eligible persons at least 50%?
Question 5: Was a sample size justification, power description or variance and effect estimates provided?
Question 6: For the analyses in this paper, were the exposure(s) of interest measured prior to the outcome(s)?
Question 10: Was the exposure(s) assessed more than once over time?
Question 12: Were the outcome assessors blinded to the exposure status of the participants?
Question 13: Was loss to follow-up after baseline 20% or less?

Supplementary figure 2. Funnel Plots for standardised beta-coefficients for systolic (A) and diastolic (B) blood pressure with white matter hyperintensity volume. SE = standard error, blue lines represent upper and lower confidence limits.

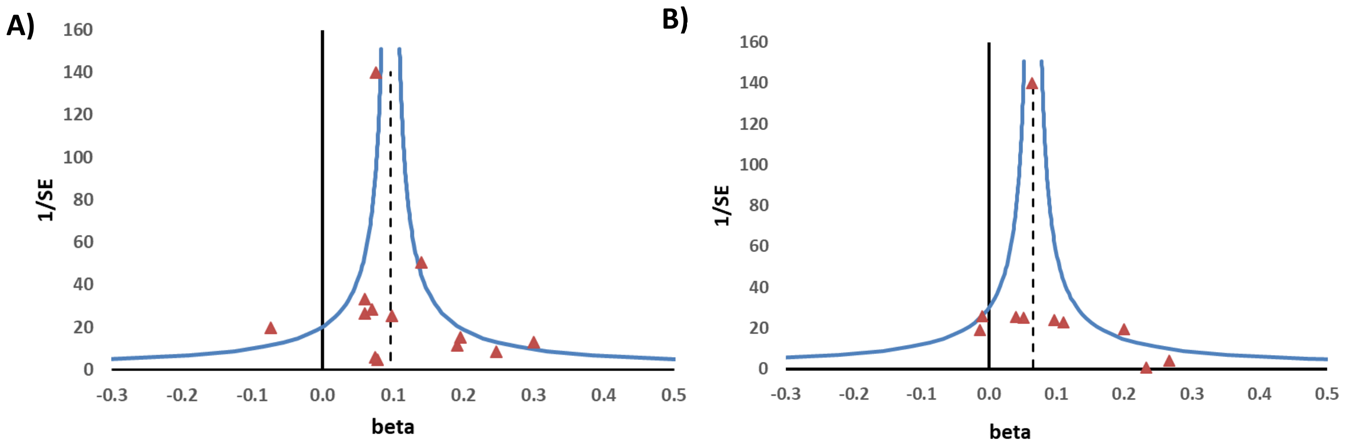

Supplementary figure 3. Unadjusted, standardised associations between SBP and DBP with WMHV. Results are combined by fixed and random effects meta-analysis, weighted by the inverse variance, with heterogeneity presented as I^2 statistics(I-sq), and the p-value for heterogeneity (p-het) determined by chi-squared testStdBeta = standardised beta; N= number;

A) SBP

Study	N	StdBeta	Cilow	Cihigh	p-value	p-Het
Alkan	164	0.206	0.061	0.351	0.005	
Cloonan	809	0.207	-0.068	0.482	0.140	
Wartolowska - concurrer	37026	0.273	0.262	0.284	<0.001	
Shimizu	133	0.273	-0.404	0.950	0.430	
Fuhrmann	667	0.350	0.115	0.585	0.004	I-sq
						0%
Fixed Effects	38799	0.273	0.262	0.284	<0.001	p-het
Random Effects	38799	0.273	0.262	0.284	<0.001	0.84

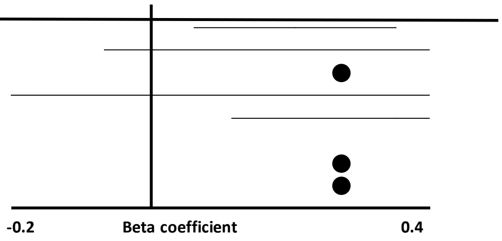

B) DBP

Study	N	StdBeta	Cilow	Cihigh	p-value	p-Het
Fuhrmann	667	-0.350	-0.546	-0.154	<0.001	
Cloonan	809	0.054	-0.864	0.972	0.910	
Wartolowska - concurrer	37026	0.083	0.071	0.095	<0.001	I-sq
						89%
Fixed Effects	38502	0.082	0.070	0.093	<0.001	p-het
Random Effects	38502	-0.099	-0.477	0.279	0.610	<0.001

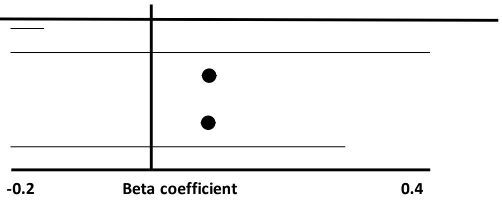

Supplementary Figure 4. Standardised associations between SBP or DBP with WMH, excluding the largest study. Results are combined by fixed and random effects meta-analysis, weighted by the inverse variance, with heterogeneity presented as I^2 statistics(I-sq), and the p-value for heterogeneity (p-het) determined by chi-squared test StdBeta = standardised beta; N= number;

A) SBP

Study	N	StdBeta	Cilow	Cihigh	p-value	p-Het
Schwartz - white	343	-0.073	-0.173	0.026	0.150	
Godin	1391	0.060	0.001	0.119	0.050	
Aribisala - concurrent	694	0.060	-0.014	0.134	0.110	
Lane	468	0.070	0.001	0.140	0.050	
White	71	0.075	-0.261	0.411	0.660	
Sander	227	0.078	-0.348	0.504	0.720	
King	1270	0.098	0.021	0.175	0.010	
Lampe	1825	0.140	0.101	0.179	<0.001	
Cui	663	0.191	0.019	0.363	0.030	
Schwartz - black	267	0.195	0.068	0.323	0.003	
Cheng	60	0.247	0.017	0.477	0.040	
Shokouhi	265	0.300	0.150	0.450	<0.001	I-sq
						64%
Fixed Effects	7544	0.102	0.079	0.126	<0.001	p-het
Random Effects	7544	0.104	0.056	0.153	<0.001	0.0011

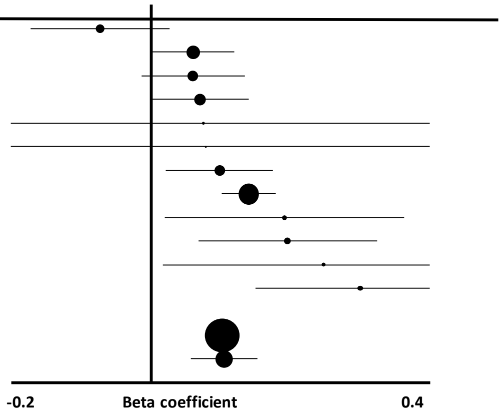

B) DBP

Study	N	StdBeta	Cilow	Cihigh	p-value	p-Het
King	1270	-0.013	-0.115	0.089	0.800	
Schwartz - white	343	-0.010	-0.087	0.066	0.790	
Aribisala - concurrent	694	0.040	-0.037	0.117	0.310	
Lane	468	0.051	-0.027	0.130	0.200	
Godin	1391	0.096	0.014	0.178	0.020	
Lampe	1825	0.110	0.024	0.196	0.010	
Shokouhi	265	0.200	0.100	0.300	<0.001	
Schwartz - black	267	0.233	-1.502	1.967	0.790	
Sander	227	0.266	-0.197	0.729	0.260	I-sq
						50%
Fixed Effects	6750	0.063	0.031	0.095	<0.001	p-het
Random Effects	6750	0.068	0.019	0.116	0.006	0.044

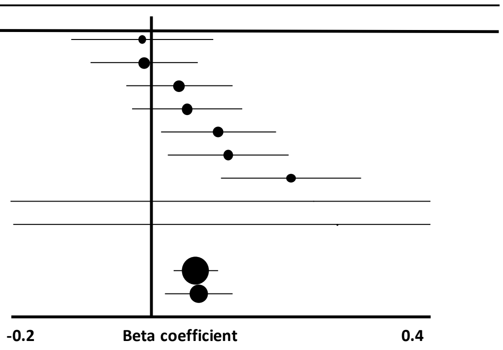

Supplementary Figure 5. Association between mean blood pressure (MBP) or pulse pressure (PP) with white matter hyperintensity volume. Results are combined by fixed and random effects meta-analysis, weighted by the inverse variance, with heterogeneity presented as I^2 statistics(I-sq), and the p-value for heterogeneity (p-het) determined by chi-squared test StdBeta = standardised beta; N= number;

A) MBP

Study	N	StdBeta	Cilow	Cihigh	p-value	p-Het
Heye	264	-0.004	-0.015	0.007	0.520	
Shi	56	0.010	-0.010	0.030	0.330	
Tsao	1118	0.070	0.011	0.129	0.020	I-sq
						70%
Fixed Effects	1438	0.001	-0.008	0.011	0.770	p-het
Random Effects	1438	0.011	-0.013	0.036	0.370	0.034

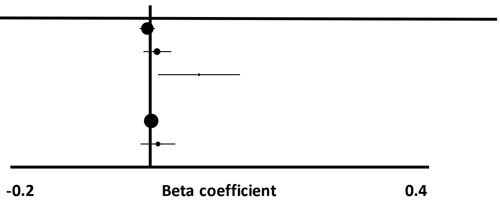

B) PP

Study	N	StdBeta	Cilow	Cihigh	p-value	p-Het
Heye	264	0.009	0.001	0.018	0.040	
Aribisala - concurrent	694	0.050	-0.024	0.124	0.190	I-sq
						13%
Fixed Effects	958	0.010	0.001	0.018	0.026	p-het
Random Effects	958	0.012	-0.009	0.034	0.260	0.28

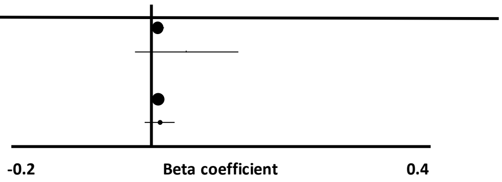

Supplementary Figure 6. Associations between DBP and WMH in studies with a mean age of less than 65 compared to greater than 65. Results are combined by fixed and random effects meta-analysis, weighted by the inverse variance, with heterogeneity presented as I^2 statistics(I-sq), and the p-value for heterogeneity (p-het) determined by chi-squared test StdBeta = standardised beta; N= number;

A) Studies in patients with a mean age <65

Study	N	StdBeta	Cilow	Cihigh	p-value	p-Het
King	1270	-0.013	-0.115	0.089	0.800	
Schwartz - white	343	-0.010	-0.087	0.066	0.790	
Lampe	1825	0.110	0.024	0.196	0.010	
Shokouhi	265	0.200	0.100	0.300	<0.001	
Schwartz - black	267	0.233	-1.331	1.796	0.770	I-sq
						72%
Fixed Effects	3970	0.064	0.019	0.108	0.005	p-het
Random Effects	3970	0.071	-0.023	0.165	0.140	0.007

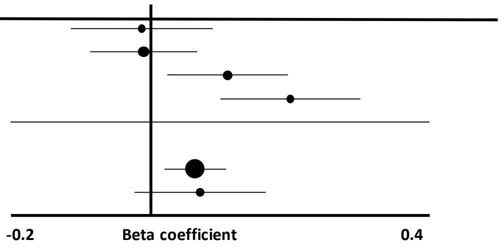

B) Studies in patients with a mean age >65

Study	N	StdBeta	Cilow	Cihigh	p-value	p-Het
Aribisala - concurrent	694	0.040	-0.037	0.117	0.310	
Lane	468	0.051	-0.027	0.130	0.200	
Godin	1391	0.096	0.014	0.178	0.020	
Sander	227	0.266	-0.197	0.729	0.260	I-sq
						0%
Fixed Effects	2780	0.063	0.018	0.108	0.007	p-het
Random Effects	2780	0.063	0.018	0.108	0.007	0.62

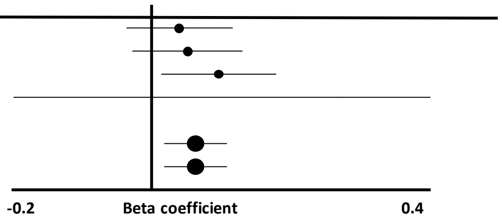

Supplementary Figure 7. Associations between DBP and WMH, stratified by time of exposure. Results are combined by fixed and random effects meta-analysis, weighted by the inverse variance, with heterogeneity presented as I^2 statistics(I-sq), and the p-value for heterogeneity (p-het) determined by chi-squared test StdBeta = standardised beta; N= number;

A) Concurrent DBP

Study	N	StdBeta	Cilow	Cihigh	p-value	p-Het
Aribisala - concurrent	694	0.040	-0.037	0.117	0.310	
Wartolowska - concurrer	37026	0.064	0.050	0.078	<0.001	
Godin	1391	0.096	0.014	0.178	0.020	
Lampe	1825	0.110	0.024	0.196	0.010	
Shokouhi	265	0.200	0.100	0.300	<0.001	
Sander	227	0.266	-0.197	0.729	0.260	I-sq
						48%
Fixed Effects	41428	0.068	0.054	0.081	<0.001	p-het
Random Effects	41428	0.090	0.048	0.132	<0.001	0.09

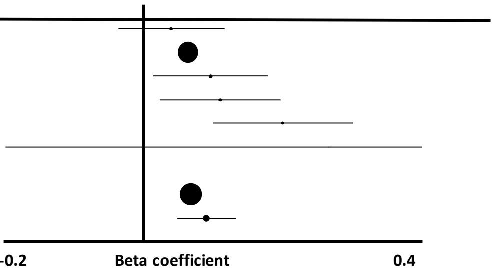

B) DBP measured > 2 years previously

Study	N	StdBeta	Cilow	Cihigh	p-value	p-Het
King	1270	-0.013	-0.115	0.089	0.800	
Aribisala - 2 years	694	0.080	0.005	0.155	0.040	
Wartolowska - past	37026	0.087	0.064	0.110	<0.001	
Lane - historic	468	0.140	0.056	0.224	0.001	I-sq
						43%
Fixed Effects	39458	0.086	0.065	0.106	<0.001	p-het
Random Effects	39458	0.082	0.040	0.123	<0.001	0.16

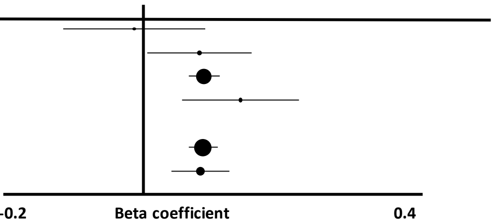

C) DBP measured >5 years previously

Study	N	StdBeta	Cilow	Cihigh	p-value	p-Het
King	1270	-0.013	-0.115	0.089	0.800	
Wartolowska - past	37026	0.087	0.064	0.110	<0.001	
Lane - historic	468	0.140	0.056	0.224	0.001	I-sq
						62%
Fixed Effects	38764	0.086	0.065	0.107	<0.001	p-het
Random Effects	38764	0.079	0.016	0.142	0.013	0.07

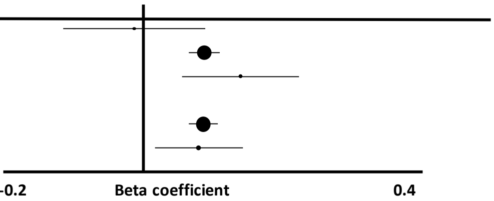

Search strategy

1. Primary Search

((“white matter hyperintensities" OR "leukoaraiosis" OR “leukoencephalopath*" OR “arteriosclerotic encephalopathy” OR "white matter hyperintensity" OR "white matter lesion*" OR "white matter disease" OR “white matter changes” OR “white matter damage” OR “ischaemic white matter disease” OR “binswanger disease” OR “hypertensive arteriopathy” OR “small vessel disease” OR “cerebral microangiopathy”) AND ("blood pressure" OR "systolic" OR "diastolic" OR "arterial pressure" OR "pulse pressure" OR "pulse wave velocity" OR "pulse transit time" OR "pulse wave transit time" OR "BP" OR "ABP" OR "pulsatile pressure” OR “cerebral blood flow” OR “cerebral blood velocity”))

2. Secondary Search Strategy

(“*Study Name”)* AND (“MRI” OR “Magnetic Resonance”)

Eg: (“Rotterdam Study” OR “Rotterdam Scan Study”) AND (“MRI” OR “Magnetic Resonance”)

References of included studies

1. Alkan E, Taporoski TP, Sterr A, von Schantz M, Vallada H, Krieger JE, Pereira AC, Alvim R, Horimoto A, Pompeia S, Negrao AB and Evans SLH. Metabolic syndrome alters relationships between cardiometabolic variables, cognition and white matter hyperintensity load. *Sci Rep*. 2019;9:4356.
2. Aribisala BS, Morris Z, Eadie E, Thomas A, Gow A, Valdes Hernandez MC, Royle NA, Bastin ME, Starr J, Deary IJ and Wardlaw JM. Blood pressure, internal carotid artery flow parameters, and age-related white matter hyperintensities. *Hypertension*. 2014;63:1011-8.
3. Cheng CY, Cheng HM, Chen SP, Chung CP, Lin YY, Hu HH, Chen CH and Wang SJ. White matter hyperintensities in migraine: Clinical significance and central pulsatile hemodynamic correlates. *Cephalalgia*. 2018;38:1225-1236.
4. Cloonan L, Fitzpatrick KM, Kanakis AS, Furie KL, Rosand J and Rost NS. Metabolic determinants of white matter hyperintensity burden in patients with ischemic stroke. *Atherosclerosis*. 2015;240:149-53.
5. Cui C, Sekikawa A, Kuller LH, Lopez OL, Newman AB, Kuipers AL and Mackey RH. Aortic Stiffness is Associated with Increased Risk of Incident Dementia in Older Adults. *J Alzheimers Dis*. 2018;66:297-306.
6. Fuhrmann D, Nesbitt D, Shafto M, Rowe JB, Price D, Gadie A, Cam CAN and Kievit RA. Strong and specific associations between cardiovascular risk factors and white matter micro- and macrostructure in healthy aging. *Neurobiol Aging*. 2019;74:46-55.
7. Godin O, Tzourio C, Maillard P, Mazoyer B and Dufouil C. Antihypertensive treatment and change in blood pressure are associated with the progression of white matter lesion volumes: the Three-City (3C)-Dijon Magnetic Resonance Imaging Study. *Circulation*. 2011;123:266-73.
8. Gutierrez J, Elkind MS, Cheung K, Rundek T, Sacco RL and Wright CB. Pulsatile and steady components of blood pressure and subclinical cerebrovascular disease: the Northern Manhattan Study. *J Hypertens*. 2015;33:2115-22.
9. Habes M, Sotiras A, Erus G, Toledo JB, Janowitz D, Wolk DA, Shou H, Bryan NR, Doshi J, Volzke H, Schminke U, Hoffmann W, Resnick SM, Grabe HJ and Davatzikos C. White matter lesions: Spatial heterogeneity, links to risk factors, cognition, genetics, and atrophy. *Neurology*. 2018;91:e964-e975.
10. Jeerakathil T, Wolf PA, Beiser A, Massaro J, Seshadri S, D'Agostino RB and DeCarli C. Stroke risk profile predicts white matter hyperintensity volume: the Framingham Study. *Stroke*. 2004;35:1857-61.
11. King KS, Chen KX, Hulsey KM, McColl RW, Weiner MF, Nakonezny PA and Peshock RM. White matter hyperintensities: use of aortic arch pulse wave velocity to predict volume independent of other cardiovascular risk factors. *Radiology*. 2013;267:709-17.
12. Lampe L, Zhang R, Beyer F, Huhn S, Kharabian Masouleh S, Preusser S, Bazin PL, Schroeter ML, Villringer A and Witte AV. Visceral obesity relates to deep white matter hyperintensities via inflammation. *Ann Neurol*. 2019;85:194-203.
13. Lane CA BJ, Nicholas JM, Sudre CH, Cash DM, Parker TD, Malone IB, Lu K, James SN, Keshavan A, Murray-Smith H, Wong A, Buchanan SM, Keuss SE, Gordon E, Coath W, Barnes A, Dickson J, Modat M, Thomas D, Crutch SJ, Hardy R, Richards M, Fox NC, Schott JM. Associations between blood pressure across adulthood and late-life brain structure and pathology in the neuroscience substudy of the 1946 British birth cohort (Insight 46): an epidemiological study. *Lancet Neurol*. 2019.
14. Maillard P, Seshadri S, Beiser A, Himali JJ, Au R, Fletcher E, Carmichael O, Wolf PA and DeCarli C. Effects of systolic blood pressure on white-matter integrity in young adults in the Framingham Heart Study: a cross-sectional study. *Lancet Neurol*. 2012;11:1039-47.
15. Sander D, Winbeck K, Klingelhofer J and Conrad B. Extent of cerebral white matter lesions is related to changes of circadian blood pressure rhythmicity. *Arch Neurol*. 2000;57:1302-7.

16. Schwartz GL, Bailey KR, Mosley T, Knopman DS, Jack CR, Jr., Canzanello VJ and Turner ST. Association of ambulatory blood pressure with ischemic brain injury. *Hypertension*. 2007;49:1228-34.
17. Shi Y, Thrippleton MJ, Blair GW, Dickie DA, Marshall I, Hamilton I, Doubal FN, Chappell F and Wardlaw JM. Small vessel disease is associated with altered cerebrovascular pulsatility but not resting cerebral blood flow. *J Cereb Blood Flow Metab*. 2020;40:85-99.
18. Shimizu A, Kokubo M, Mitsui T, Miyagi M, Nomoto K, Murohara T, Toba K and Sakurai T. Left ventricular diastolic dysfunction is directly associated with cerebral white matter lesions in elderly patients. *Geriatr Gerontol Int*. 2015;15 Suppl 1:81-2.
19. Shokouhi M, Qiu D, Samman Tahhan A, Quyyumi AA and Hajjar I. Differential Associations of Diastolic and Systolic Pressures With Cerebral Measures in Older Individuals With Mild Cognitive Impairment. *Am J Hypertens*. 2018;31:1268-1277.
20. Tsao CW, Himali JJ, Beiser AS, Larson MG, DeCarli C, Vasan RS, Mitchell GF and Seshadri S. Association of arterial stiffness with progression of subclinical brain and cognitive disease. *Neurology*. 2016;86:619-26.
21. Vesperman CJ, Pozorski V, Dougherty RJ, Law LL, Boots E, Oh JM, Gallagher CL, Carlsson CM, Rowley HA, Ma Y, Bendlin BB, Asthana S, Sager MA, Hermann BP, Johnson SC, Cook DB and Okonkwo OC. Cardiorespiratory fitness attenuates age-associated aggregation of white matter hyperintensities in an at-risk cohort. *Alzheimers Res Ther*. 2018;10:97.
22. Wartolowska KA and Webb AJS. Midlife blood pressure is associated with the severity of white matter hyperintensities: analysis of the UK Biobank cohort study. *Eur Heart J*. 2020.
23. White WB, Wolfson L, Wakefield DB, Hall CB, Campbell P, Moscufo N, Schmidt J, Kaplan RF, Pearlson G and Guttmann CR. Average daily blood pressure, not office blood pressure, is associated with progression of cerebrovascular disease and cognitive decline in older people. *Circulation*. 2011;124:2312-9.
24. White WB, Jalil F, Wakefield DB, Kaplan RF, Bohannon RW, Hall CB, Moscufo N, Fellows D, Guttmann CRG and Wolfson L. Relationships among clinic, home, and ambulatory blood pressures with small vessel disease of the brain and functional status in older people with hypertension. *Am Heart J*. 2018;205:21-30.
25. Wolfson L, Wakefield DB, Moscufo N, Kaplan RF, Hall CB, Schmidt JA, Guttmann CR and White WB. Rapid buildup of brain white matter hyperintensities over 4 years linked to ambulatory blood pressure, mobility, cognition, and depression in old persons. *J Gerontol A Biol Sci Med Sci*. 2013;68:1387-94.
